# Elimination of Composition Segregation in 33Al–45Cu–22Fe (at.%) Powder by Two-Stage High-Energy Mechanical Alloying

**DOI:** 10.3390/ma15062087

**Published:** 2022-03-11

**Authors:** Serguei Tikhov, Konstantin Valeev, Svetlana Cherepanova, Vladimir Zaikovskii, Aleksei Salanov, Vladislav Sadykov, Dina Dudina, Oleg Lomovsky, Sergey Petrov, Oleg Smorygo, Amol Gokhale

**Affiliations:** 1 Boreskov Institute of Catalysis SB RAS, Lavrentyev Ave. 5, 630090 Novosibirsk, Russia; valeev@catalysis.ru (K.V.); svch@catalysis.ru (S.C.); viz@catalysis.ru (V.Z.); salanov@catalysis.ru (A.S.); sadykov@catalysis.ru (V.S.); 2 Lavrentyev Institute of Hydrodynamics SB RAS, Lavrentyev Ave. 15, 630090 Novosibirsk, Russia; dina1807@gmail.com; 3 Institute of Solid State Chemistry and Mechanochemistry SB RAS, Kutateladze Str. 18, 630128 Novosibirsk, Russia; lomov@solid.nsc.ru (O.L.); petrov@solid.nsc.ru (S.P.); 4 O.V. Roman Powder Metallurgy Institute, National Academy of Sciences of Belarus, 41 Platonov Str., 220005 Minsk, Belarus; smorygo@tut.by; 5 Indian Institute of Technology Bombay, Powai, Mumbai 400076, India; gokhale.iitb@gmail.com

**Keywords:** mechanical alloying, powder, Cu–Fe alloy, Cu–Fe–Al alloy, catalysts

## Abstract

In the present work, complex powder alloys containing spinel as a minor phase were produced by mechanical alloying in a high-energy planetary ball mill from a 33Al–45Cu–22Fe (at.%) powder blend. These alloys show characteristics suitable for the synthesis of promising catalysts. The alloying was conducted in two stages: at the first stage, a Cu+Fe powder mixture was ball-milled for 90 min; at the second stage, Al was added, and the milling process was continued for another 24 min. The main products of mechanical alloying formed at each stage were studied using X-ray diffraction phase analysis, Mössbauer spectroscopy, transmission electron microscopy, and energy-dispersive spectroscopy. At the end of the first stage, crystalline iron was not found. The main product of the first stage was a metastable Cu(Fe) solid solution with a face-centered cubic structure. At the second stage, the Cu(Fe) solid solution transformed to Cu(Al), several Fe-containing amorphous phases, and a spinel phase. The products of the two-stage process were different from those of the single-stage mechanical alloying of the ternary elemental powder mixture; the formation of undesirable intermediate phases was avoided, which ensured excellent composition uniformity. A sequence of solid-state reactions occurring during mechanical alloying was proposed. Mesopores and a spinel phase were the features of the two-stage milled material (both are desirable for the target catalyst).

## 1. Introduction

Ternary Al–alloys, such as Al–Cu–Fe mixed with spinel, are among the important materials to develop ceramometal (‘cermet’) catalysts. The effectiveness of a catalyst increases with specific surface area (as in porous materials), the number of active centers, and the density of the catalyst material. Ceramometals are attractive in heterogeneous catalysis since their surfaces possess a higher number of catalytically active centers than oxides, and their density is about twice the density of porous oxide systems [1]. Another desirable characteristic of catalysts is their stability in reaction media, often displayed by spinels, such as CuFe_2_O_4_ [2]. However, the formation of the CuFe_2_O_4_-type spinel needs to be preceded by the formation of a copper-iron alloy (which is not possible by conventional processing because of no mutual solubility) before aluminum is introduced.

Most studies on Al–Cu–Fe alloys were aimed at the synthesis of Al_63-70_Cu_20-25_Fe_10-12_ quasicrystals of icosahedral structure [3,4,5,6,7] for a variety of applications: antimicrobial agents [8], decomposition of hazardous materials [9], carbon nanotube growth catalysts [10], magnetic materials [4,11], anodes in lithium batteries [12], fillers with ultralow wear [13], and catalysts in steam reforming of methanol [14,15]. Moreover, nanostructured powder alloys are becoming popular in traditional heterogeneous catalysis [16,17], e.g., in hydrogenation reactions of CO (CO_2_) [18], synthesis of carbon fibers [19], decomposition of chlorine-containing hydrocarbons [20,21], decomposition of polymers [22], and in steam and dry reforming [23].

As mentioned earlier, for catalysis, the specific surface area must be high, which implies that the size of the powder particles and nano-aggregates must be small, which can be obtained by processes, such as self-propagating high-temperature synthesis [16,17,18]. However, even a short high-temperature exposure can be detrimental, leading to aggregate coarsening, which requires additional processing to increase the specific surface area of the powders [17]. Another possibility to produce fine powders of multi-component alloys is through mechanical alloying [24]. This method is widely used for the preparation of nanostructured Cu–Fe–Al alloys [5] and porous nanocomposite ceramometals. In general, the mechanical properties of a nanocomposite material depend upon the process of its preparation [25]. The properties of ceramometal catalysts have been shown to depend on the conditions of mechanical alloying of the powder mixtures. Their applications in low-temperature water–gas shift reactions have been reported [26,27].

In mechanical alloying, metal powders are subjected to high-energy mechano-chemical treatment to ensure uniform mixing of the constituents at the atomic level leading to the formation of solid solutions and intermetallic compounds. This is required for the formation of closely dispersed catalytically active binary and ternary spinel phases during further low-temperature oxidation. Hydrothermal treatment and calcination at moderate temperatures (500–600 °C) of the powders ensure the formation of a nano-porous active material consisting of the oxide and the metallic phases. Nanostructured alloys with high concentrations of crystalline imperfections prepared by mechanical alloying are preferred over well-crystallized phases [1,28].

Al_63-70_Cu_20-25_Fe_10-12_ quasicrystalline powders with a uniform distribution of metallic components were obtained by single-stage mechanical alloying followed by heat treatment [29,30]. Due to a high hardness, these phases were used for reinforcing aluminum alloys [31,32].

The process of single-stage mechanical alloying of elemental powders with overall composition 33Al–45Cu–22Fe by high-energy ball milling has been reported earlier [33]. The formation of Cu–Al phases were shown to follow the sequence Al_2_Cu → Al_4_Cu_9_ → Cu(Al) solid solution. The weight ratio between Al and Fe calculated from XRD quantitative analysis data did not correspond to the real content of these metals in the initial powder mixture. No phases containing both Fe and Cu were found in the mechanically alloyed material or after further oxidative treatments [33,34]. However, prolonged mechanical alloying of Cu and Fe powders was shown to result in the formation of an amorphous metastable ‘phase’ with non-uniform distribution of the elements [35,36]. This may be a promising intermediate phase for obtaining Cu–Fe–Al alloys, in which Cu and Fe are thoroughly intermixed (at the atomic level), containing a higher number of active spinel centers. Combining the ideas presented above, it can be expected that a powder in the ternary Cu–Fe–Al system with atomically intermixed Cu and Fe can be achieved via a two-stage mechano-chemical alloying process. In this approach, the role of the first stage is to form a Cu–Fe pseudo-alloy, which is essential for binding aluminum with both elements for forming metastable ternary phases [37] that decompose in the form of finely dispersed and uniformly distributed inclusions.

The present work reports the synthesis of a 33Al–45Cu–22Fe alloy powder by two-stage mechanical alloying for the first time. The alloy possesses characteristics that could not be achieved by single-stage milling of the three elemental powders. Both stages of alloying were carried out in a high-energy planetary ball mill. Changes in the microstructure at the first stage of alloying (in a binary Cu–Fe mixture) and at the second stage (after adding Al to the products of the first stage of alloying) were studied and described. A large amount of amorphous states was monitored.

## 2. Materials and Methods

### 2.1. Materials

An aluminum powder with platelet-like particles and the particle diameter of several tens of microns (PAP-2, GOST 5494-95, SUAL-PM Ltd., Shelekhov, Russia) [33], an electrolytic copper powder with a dendritic particle shape (PMS-1, GOST 4960-75, Uralectromed, Verhnyaya Pyshma, Russia) [1], and a high-purity iron powder (PANREAC-141901, Panreac Quimica SLU, Barcelona, Spain) were used. The morphologies of the starting powders were presented earlier in [27,28].

### 2.2. Mechanical Alloying

In the present work, mechanical alloying was conducted in two stages. The first stage involved high-energy ball milling of Cu and Fe elemental powders at the atomic ratio of Cu:Fe = 67:33. The powder mixture was milled in an APF-type planetary ball mill equipped with two steel-milling vials. The volume of the milling vial was 1 *L*. Steel-milling balls, 5 mm in diameter, were used. Milling was conducted in air at an acceleration of the balls of 200 m s^−2^ and a ball-to-powder weight ratio of 20:1.

**Figure 1:**
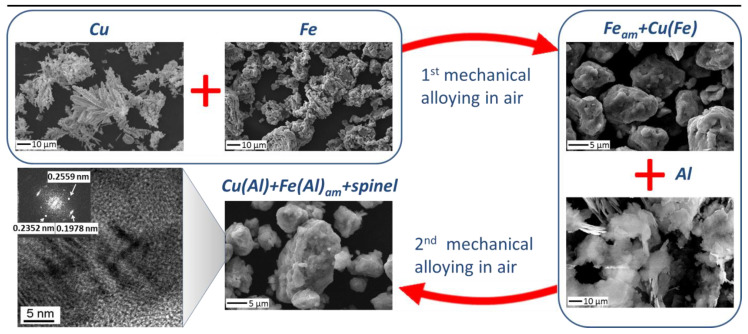
Scheme of the preparation procedure.

The milling time of the Cu+Fe powder mixture varied from 15 min to 90 min with 15 min increments. The milling process was interrupted after the given periods, and aluminum powder was added into the vials such that the atomic ratio Al:Cu:Fe was maintained at 33:45:22. The second-stage milling was carried out for 24 min irrespective of the first-stage milling time. Ethanol was added into the vials at both milling stages 30 s before the stage completion (2 mL). Ethanol was introduced to eliminate the sticking of the powders to contacting surfaces of the vials and balls. The scheme of the powder preparation procedure is presented in Figure 1.

### 2.3. Methods

XRD patterns of the alloyed powders in the interval 2θ = 20–97° were recorded using a D8 ADVANCE X-ray diffractometer (Bruker AXS, Karlsruhe, Germany) equipped with a one-dimensional Lynx-Eye detector under Cu Kα radiation with a step of 0.05° and 3 s/step counting time. The quantitative analysis of the phases was performed in the 2θ range of 37–55° using Rietveld refinement in the TOPAS 4.2 software (Bruker AXS). TOPAS software was applied to determine an average crystallite size, taking into account the instrumental peak broadening. An XRD pattern of corundum (SRM676), a reference sample, was used to estimate the peak broadening applying the approximation method by convolution of Lorentz and Gauss functions. The contents of Al in Cu(Al) solid solutions were calculated using Vegard’s law; it was assumed that no iron was present in the solid solution.

The powder morphology and microstructure were studied by scanning electron microscopy (SEM) using a JSM 6460SV microscope (JEOL Ltd., Tokyo, Japan) equipped with an energy-dispersive spectroscopy unit INCA EDX (Oxford Instruments, London, UK). Powders were spread over a substrate to observe the surface morphology or mounted in a resin binder and polished.

Transmission electron microscopy (TEM) micrographs were received on a JEM-2010 and a JEM-2200FS (JEOL Ltd., Tokyo, Japan) microscopes). Th high-angle annular dark-field scanning transmission electron mode (HAADF STEM) and energy-dispersive X-ray spectroscopy (EDX) were applied. Al Kα (E = 1.486 keV), Fe Kα (6.938 keV), and Cu Kα (E = 8.040 keV) radiations were used for elemental mapping by EDX in the STEM mode. Semi-quantitative analysis of very small (~1 nm) areas was possible. The powder samples for TEM studies were suspended in ethanol and ultrasonicated at 35 kHz. Then the suspension was poured onto a holey carbon film substrate mounted on a Mo grid and dried at 20 °C.

Mössbauer spectroscopy (MS, MTA KFKI, Budapest, Hungary) was carried out using a spectrometer with ^57^Co(Rh) source at a constant acceleration mode. The measurements were conducted at room temperature.

The specific surface area was calculated by the Brunauer–Emmett–Teller (BET, ASAP-2400 instrument, Micromeritics, Norcross, USA) method from the adsorption isotherms of argon.

## 3. Results

### 3.1. Mechanical Alloying of Cu and Fe

**Figure 2:**
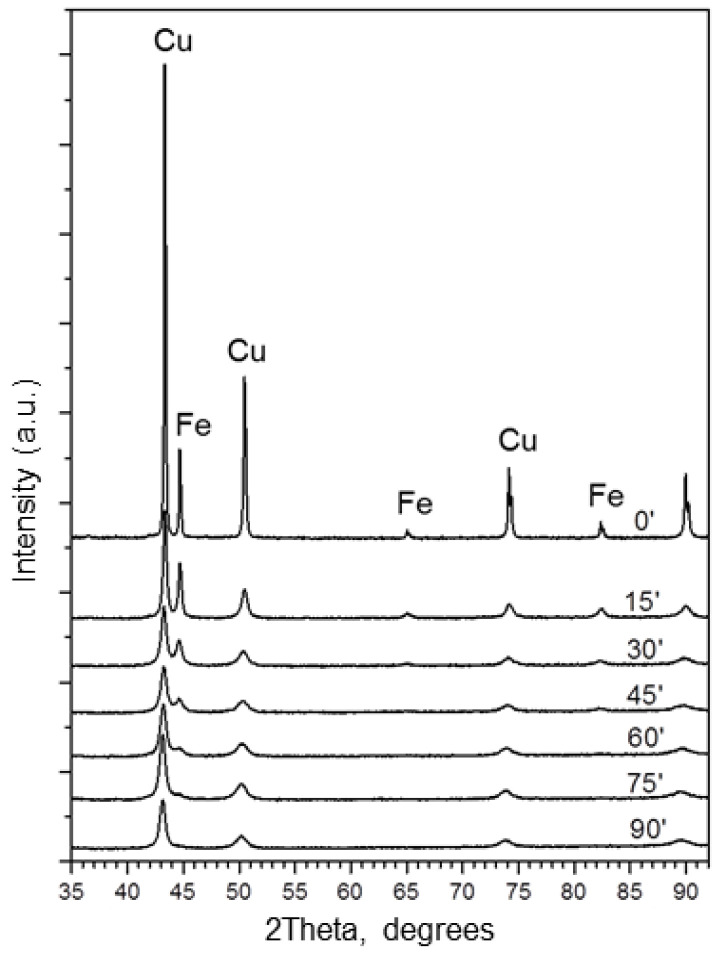
XRD patterns of the 67Cu–33Fe powders ball-milled for 0 min, 15 min, 30 min, 45 min, 60 min, 75 min and 90 min. “Cu”—Cu(Fe) fcc solid solution; “Fe”—α-Fe bcc.

The XRD patterns of the Cu+Fe powder mixtures after milling for different times are shown in Figure 2. As the milling time increases, the peaks of copper become broader, while those of iron decrease in intensity and subsequently disappear.

The evolution of the lattice parameters of the phases, crystallite sizes, and phase ratios in the mixture with the milling time is presented in Table 1. The fcc solid solution lattice parameter increases from 3.615 Å (before milling) to 3.634 Å (after 90 min of milling). The lattice parameter of iron does not change significantly. The crystallite sizes of the phases decrease first and then level off at ~10 nm after 45 min of milling. After 90 min of milling, the peaks corresponding to iron are not visible in the XRD patterns indicating the absence of free crystalline iron in the alloy. Similar observations have been reported for mixtures containing 10–45 at.% of iron milled for 75 h [35,36]. For a mixture with a composition close to that taken in the present work, an increase in the lattice parameter of the fcc structure relative to pure copper was recorded after prolonged milling. The reported lattice parameter (~3.643 Å) [35] was larger than the value measured in the present work for the alloy milled for 90 min.

- The ICDD data the observed phases are given.

**Figure 3:**
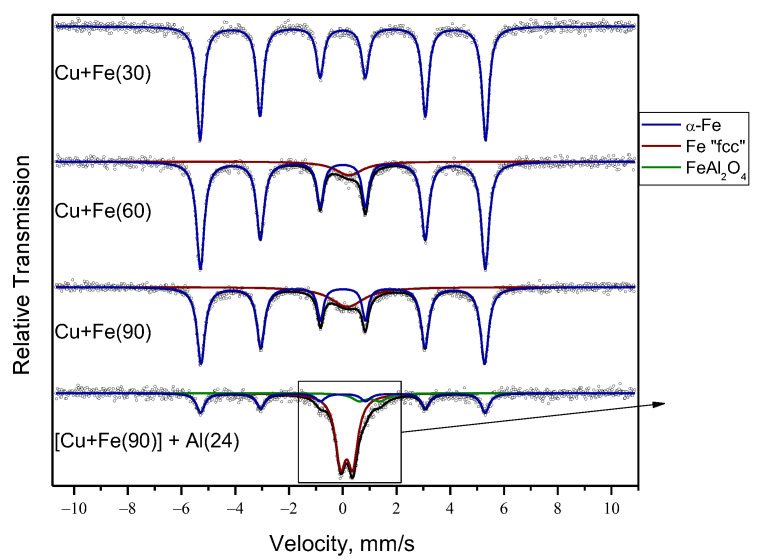
Mössbauer spectra of the products of MA Cu+Fe and (Cu+Fe) + Al. In the inset, an enlarged view of a part of the spectrum is presented.

Table 2 and Figure 3 show the results of MS of the mechanically alloyed powders. The concentration of α-Fe decreases with increasing milling time of the Cu+Fe powder mixture. Furthermore, broadening of the Fe lines is observed: from 0.30 ± 0.1 mm·s^−1^ in pure iron to 0.36 ± 0.1 mm·s^−1^ in the Cu+Fe mixture milled for 90 min. After 60 min, another state of iron appears; it is significantly different by the value of the isomer shift and is designated as Fe “fcc”. Considering the results of XRD, it can be assumed that this state of iron is related to the incorporation of iron atoms into the fcc lattice of copper. It is likely that this is a mixture of states ranging from single atoms of iron to their clusters distributed in the copper lattice [35,36], as, in our case, an asymmetric doublet mentioned in Ref. [36] does not have sufficient time to form.

There is an obvious discrepancy between the results of XRD and MS for mixtures milled for 30 min and 90 min (Table 1 and Table 2). According to the results of XRD, in the sample milled for 90 min, the concentration of α-Fe is close to zero. The MS analysis of this mixture indicates that the major part of iron is in the α-Fe state. In the sample milled for 30 min, MS indicates no “fcc” Fe (Table 2). This can be due to fluorescence during the XRD analysis, leading to partial absorption of X-rays in Fe instead of diffraction. For example, in a blend of Ni and Fe (50:50 at.%), with the use of CuK_α_ radiation, only 60% of Fe is “visible” [38]. Thus, the Cu:Fe ratio in Table 1 should be treated as “apparent” only.

**Figure 4:**
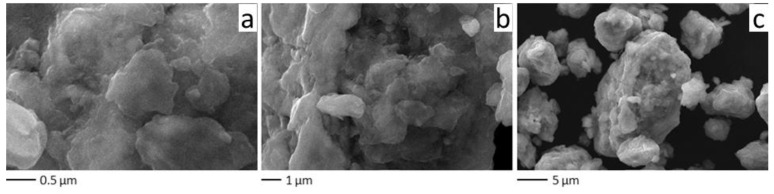
SEM images of the powder particles formed after 90 min of milling of the 67Cu–33Fe mixture at different magnifications (**a**) ×30,000 (**b**) ×10,000 and (**c**) ×3,000.

The SEM analysis showed that the Cu+Fe powder consists of aggregates of irregular shape ranging from 1 μm to 10 μm after 90 min of milling (Figure 4). The aggregate is composed of smaller round-shaped particles separated by pores between them. The surface of the aggregates is rough, showing asperities (hillocks) ~0.1 μm in size. The nature of the aggregate morphology suggests that particles of higher hardness (iron) are embedded into a softer matrix (copper-based solid solution).

**Figure 5:**
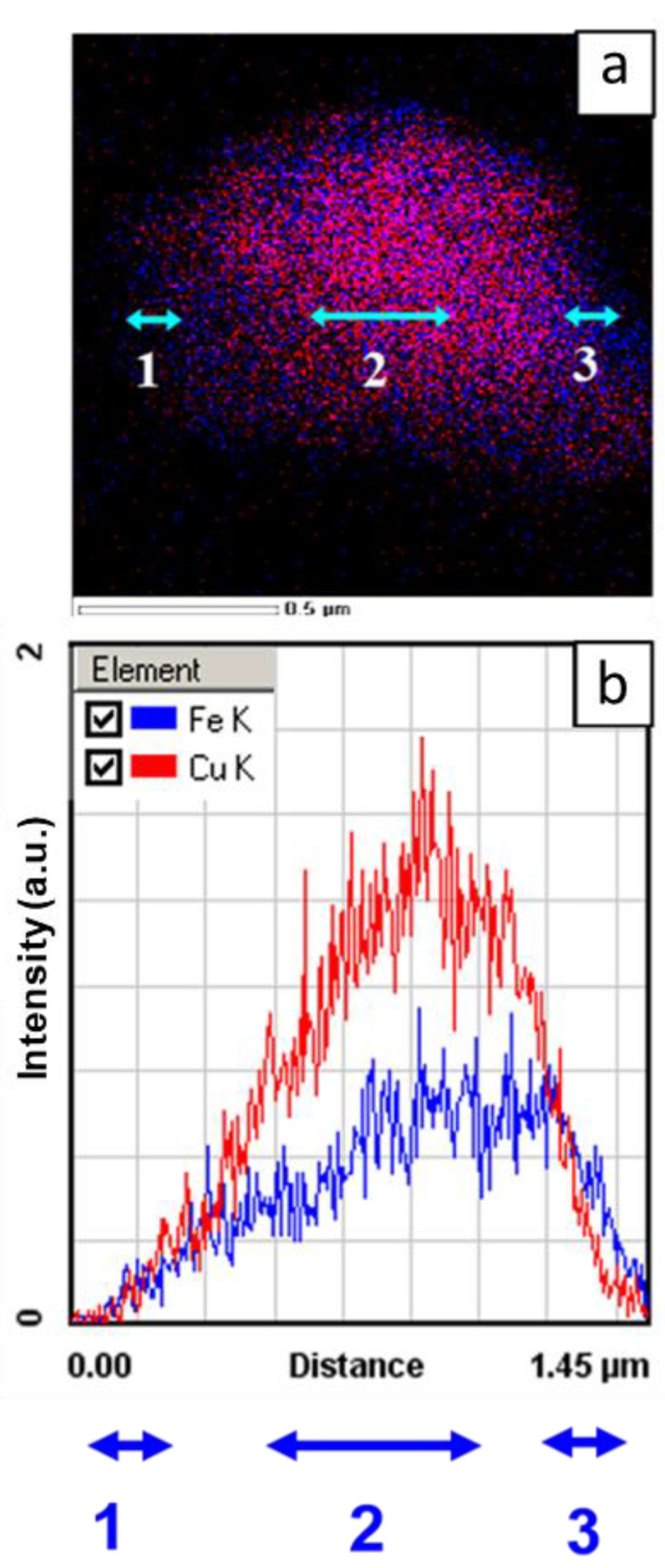
Microchemical analysis of a Cu+Fe particle aggregate after 90 min of milling (**a**) superposition of the EDX maps of combining Fe and Cu showing the flat projection of the element distributions inside the particle (**b**) intensities of Fe K and Cu K signals along the directions marked in (**a**) (averaged over the width of the band).

EDX maps of Fe and Cu in the particle (Figure 5) show that the distribution of the elements is non-uniform. The intensity of signals from the particle edges is lower due to the smaller thickness of those regions. The results of the quantitative analysis are given in Table 3. In the interior of the particles, the concentration of copper is higher than the nominal concentration. In the edges, the concentration of iron is higher than the nominal concentration (Table 3).

**Figure 6:**
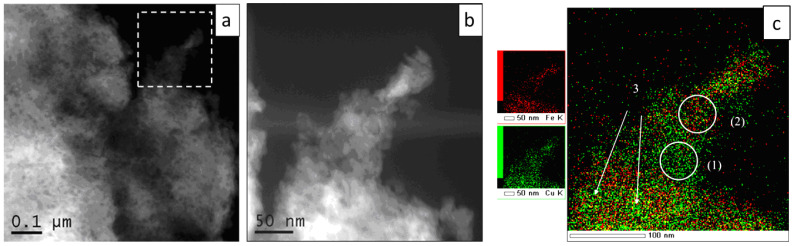
(**a**) STEM-HAADF image of a Cu+Fe aggregate (90 min of milling), (**b**) a higher magnification image, (**c**) superposition of the EDX maps for the area shown in (**b**): (1) Cu–rich areas, (2) Fe-rich areas, (3) layers of iron between copper particles.

The size of mesopores present in the particles ranges from 5 nm to 10 nm (Figure 6a). These pores can form when the particles reduce their size at the beginning of milling and aggregate later. A similar particle fragmentation followed by aggregation has been observed in ref. [33].

It can be seen at higher magnification that the particles’ edges are not smooth after 90 min of milling (Figure 6b). The distribution of the elements in such regions is rather non-uniform (Figure 6c). Areas with an excess of copper or iron can be detected; areas having a composition close to the nominal value are also found (Table 4). Thin layers of iron are seen between the copper particles (Figure 6c, marked by arrows (3)). This region corresponds to a metastable solid solution of iron in copper. Area (1) corresponds to Cu–rich crystallites, while area (2) corresponds to Fe-rich crystallites. This interpretation of the microscopy data agrees with the results of XRD and MS.

To summarize, in the first stage of mechanical alloying, the average size of copper and iron crystallites decreases from >100 nm down to 10 nm. Also, even finer crystallites of iron form and become embedded between the crystallites of copper. Upon further milling, iron partially dissolves in copper to form a solid solution, which is reflected by an increase in the lattice parameter of copper. A portion of iron gets dissolved in the copper lattice, while the remaining iron exists as clusters of iron atoms. This results in the formation of a mixture consisting of a complex Cu(Fe) + Fe cluster nanocomposite and α-Fe phase “invisible” due to X-ray fluorescence. The rate of alloying in the present study was found to be faster compared with low-energy milling reported previously [20,21].

### 3.2. Mechanical Alloying of a Mixture of Ball-Milled (Cu+Fe) and Newly Added Al

To prepare a Cu–Fe–Al precursor for the catalyst synthesis, the mechanically alloyed mixture of Cu+Fe (milling time 90 min) was further milled with an addition of aluminum. Milling was continued until the powder changed its color from silvery to black (24 min after the Al powder was added). The ternary mixture had a composition of 33Al–45Cu–22Fe (at.%).

**Figure 7:**
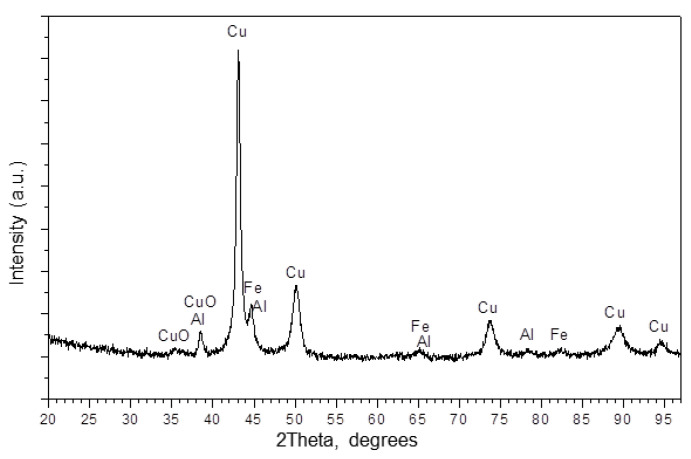
XRD pattern of the 33Al–45Cu–22Fe ((Cu+Fe) + Al) powder milled for 24 min. “Cu”—Cu–Al fcc solid solution; “Fe”—α-Fe bcc variant having a slightly higher lattice constant.

As seen in the XRD pattern of the (Cu+Fe) + Al mixture (Figure 7), the major phase in the product of co-milling of the Cu+Fe mixture with Al is a crystalline solid solution based on Cu with a lattice parameter 3.636 Å (ICDD, PDF#04-0836). Other (minor) phases are aluminum (ICDD, PDF#04-0787), α-Fe (2.871 Å) (ICDD. PDF#06-0696), and tenorite (ICDD, PDF#05-0661). After milling the mixture with Al, the lattice parameter of copper did not change significantly (Table 1, 90 min). A small increase in the lattice parameter of iron relative to the initial α-Fe is observed. The presence of tenorite is due to the oxidation of copper during milling. Of particular importance is the presence of 5 wt.% of the α-Fe phase in the (Cu+Fe) + Al mixture (note that, by the end of the first alloying stage, no crystalline Fe-based phase was present). Other crystalline Fe-containing compounds were not detected in the (Cu+Fe) + Al mixture.

MS allowed further characterizing the Fe-containing compounds in the ternary alloy obtained at the end of the second stage. The MS analysis showed that the ternary alloy contains α-Fe (32%) along with iron, the parameters of which correspond to symmetric doublet close to Fe “fcc” (59%). However, for that state of iron, there is quadrupole splitting not observed for Fe “fcc” (Table 2). The parameters of this doublet are close to those present in the spectra of Fe–Al alloys [39,40]. According to MS, milling of the ternary mixture significantly reduces the content of α-Fe in the mixture as compared with the mixture obtained at the first alloying stage (Cu+Fe, 90 min). This can be due to the interaction of iron in both α-Fe and Fe “fcc” states present in the binary alloy with aluminum resulting in the formation of X-ray amorphous Fe–Al and Cu–Fe–Al phases. In addition, Fe^2+^ is detected in the ternary alloy, which can be attributed to the spinel phase (Table 2). The quadrupole splitting of this state has been described in reference [41]. A larger isomer shift observed for this state may be related to partial occupancy of the octahedral spinel sites by Fe^2+^. The spinel phase is a product of the interaction of the alloy with the oxygen of the air and can be described as X-ray amorphous since it is not detected by the XRD analysis (PDF#340192).

The results of the XRD and MS studies lead to a conclusion that the Cu–based solid solution is not a simple solid solution of iron in copper, as the MS spectrum is different from that of the “fcc” phase in the Fe+Cu system. Presumably, the solid solution is a solution of aluminum in copper, and a change in its lattice parameter is caused by the dissolution of aluminum in copper (5 at.%).

**Figure 8:**
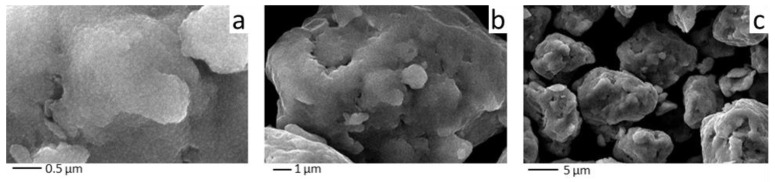
SEM images of the powder particles formed after 24 min of milling of the ternary (Cu+Fe) + Al mixture at different magnifications (**a**) ×30,000 (**b**) ×10,000 and (**c**) ×3000.

The SEM images show that, at low magnification, the shape of the particles is similar to that shown in Figure 4. At a higher magnification, the surface morphology of the ternary alloy particles is quite different from that of the binary alloy particles. The (Cu+Fe) + Al particle surface is smoother; some aggregates acquire a platelet shape (Figure 8).

**Figure 9:**
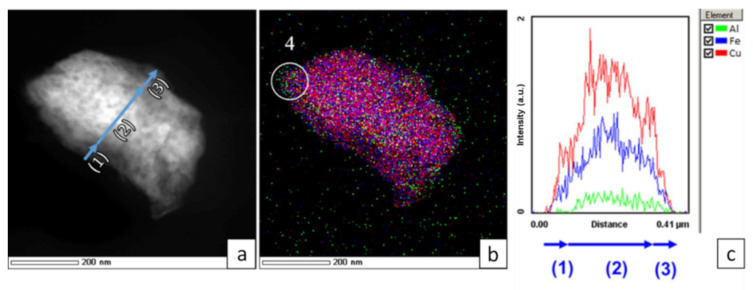
(**a**) STEM-HAADF image of a particle aggregate (Cu+Fe) + Al (24 min), (**b**) superposition of the EDX maps combining Fe, Cu and Al signals showing the element distributions in the 33Al–45Cu–22Fe particle (**a**,**c**) intensity of Fe K, Cu K and Al K signals along the line shown in (**a**). (1)–(3) different regions of particle at (**a**).

The TEM analysis shows that the ternary alloy particles contain mesopores (Figure 9a) similar to those observed in the particles of the binary alloy. A lower intensity at the edges is again related to a smaller thickness of the particle in those areas. The intensities of all the elements are seen to pass through the maximum, presumably due to a lower density of the particles near the edge. The distribution of Fe and Cu in the ternary alloy particles is more uniform (Figure 9b, Table 5) than in the binary alloy particles (Table 3). In some areas, the concentration of aluminum is only 3.2–8.1 at.%, which is much lower than the nominal concentration (Table 3). In other areas, the concentration of aluminum is higher (Figure 9b, point 4—edge of the particle, Table 5). These results indicate a non-uniform distribution of the metals in the alloy at the nano-level.

**Figure 10:**
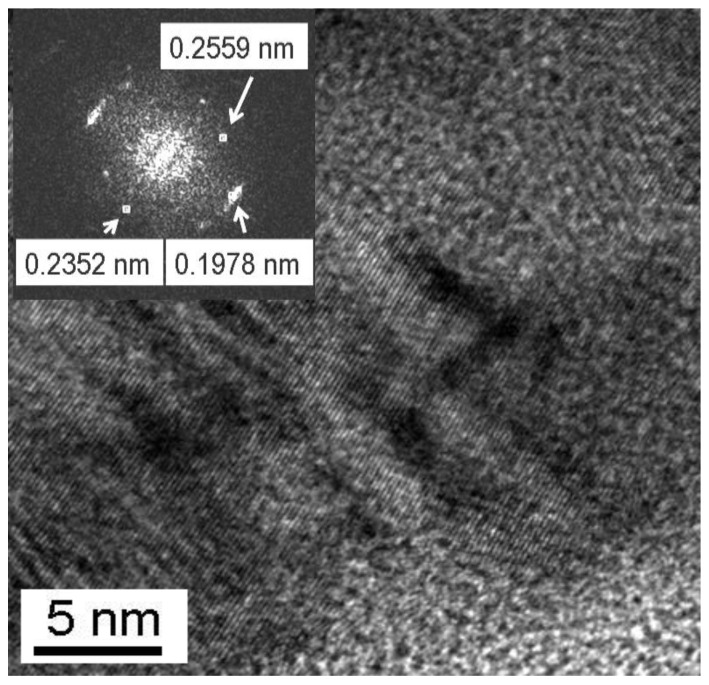
HRTEM image and FFT (inset) of the (Cu+Fe) + Al alloy particle.

A high-resolution TEM (HRTEM) image of a ternary alloy particle is presented in Figure 10. Points visible on the Fast Fourier Transform (FFT) of the image (inset) indicate the periodicity of the lattice and the presence of lattice spacing corresponding to a metallic phase Cu(Al) (d_111_ ≈ 0.2 nm) and the oxide phases Fe_3_O_4_ (d_311_ ≈ 0.25 and d_222_ ≈ 0.24 nm) and FeAl_2_O_4_ (d_111_ ≈ 0.24 nm) detected by MS. Wide dark strips are indicative of the presence of lattice microstrain. The Fe(Al) solid solution is not detected by the XRD, and, therefore, can be considered X-ray amorphous. The iron particles may be partially covered with a Cu(Al) solid solution and oxide phases of spinel-type.

## 4. Discussion

### 4.1. Phase Composition and Microstructure of the Mechanically Alloyed Cu+Fe Powder Mixture

The Cu–Fe system is immiscible because of the difference in lattice structures of the metals (fcc for Cu and bcc for α-Fe) and has a positive heat of mixing [42]. However, when the temperature reaches 920 °C, α-Fe transforms into γ-Fe having fcc lattice, which enables the formation of metastable solid solutions. Metastable copper-based solid solutions containing up to 22 at.% of iron were obtained by quenching from the melt [43]. In addition, Cu–Fe solid solutions with 57 at.% of copper were obtained by quenching from the vapor phase [44].

During mechanical alloying, the temperature of the particles increases, which facilitates the formation of a solid solution between Cu and Fe. In Ref. [45], a Fe-Cu alloy was obtained by mechanical alloying of mixtures containing an excess of iron. In several publications, Cu–Fe alloys were obtained by mechanical alloying of Cu–rich mixtures. The results have been summarized in [46]. From many reports, it is seen that a metastable solid solution of iron in copper can be formed in Cu–rich mixtures as a result of mechanical alloying [35,36,47,48,49,50,51,52,53,54]. These solid solutions are of fcc structure. The concentration of iron in the solid solution and the concentration of copper in the mixture, at which the solid solution forms, depending on the processing conditions. Reflections of the bcc phase were no longer visible in the XRD patterns of the mixtures after 400 h [49], 150 h [48], 65 h [35], 24 h [50,53], 16 h [51], or 4 h [52] of mechanical alloying. If high-energy milling is used, the peaks of iron disappear from the XRD patterns after 30 min of treatment [47].

In most publications, an increase in the lattice parameter of copper is observed, while the sextet disappears from the Mössbauer spectra of the mechanically milled mixtures. These effects are attributed to the dissolution of iron in fcc copper. However, the states of iron in the lattice of copper can be different. In Ref. [52], in a Fe30Cu80 mixture milled at a ball-to-powder weight ratio of 5:1 for 24 h, iron clusters were not found in the fcc structure. In the MS spectrum of the Fe20Cu80 mixture milled for 16 h, only a non-magnetic doublet was detected [51]. In [49], the doublet was seen in the spectrum of Fe20Cu80 milled for 600 h. This doublet is observed when Fe atoms are surrounded by at least one Fe atom but less than six small Fe clusters. The authors of [36] concluded on the formation of iron clusters of similar shape in the powder mixture milled for 75 h. At a concentration of iron of 25 at.% or lower, a broadened doublet with distribution was found in the spectrum of Fe30Cu70. In the spectra of samples milled for shorter periods, there was a sextet characteristic of α-Fe. At a lower concentration of iron, only a singlet was detected, caused by the isolated iron atoms [36].

In the MS of our samples, we did observe a state different from α-Fe, which we ascribed to a solid solution of Fe in the fcc structure of Cu. It should be mentioned that there is a difference between our results and the literature data. First, the lines of metallic iron disappear from the XRD patterns faster than in investigations of other authors. According to [47], this is a feature of the high-energy ball milling process realized in an APF mill. Second, the disappearance of iron lines from the XRD patterns is not accompanied by the disappearance of the α-Fe state from the MS. We believe that high-energy milling results in the formation of X-ray amorphous iron at the first stage (during milling of the Cu+Fe mixture). The iron is concentrated on the surface of the alloy particles (Figure 4, Table 3). It is only after longer milling durations that iron interacts with copper to form a metastable solid solution:

Fe → Cu → Cu(Fe) (1)

Part of α-Fe remains unreacted.

The formation of Cu(Fe) can occur at the boundary between copper and iron crystallites when this boundary becomes two inter-atomic distances wide, which facilitates the formation of new phases [47]. The information extracted from Figure 6c speaks in favor of this hypothesis: in the Cu–Fe alloy particles, on a 10-nm scale, both Cu–rich and Fe-rich regions are present. It should be noted that, when measured at a lower magnification, the distribution of the elements appears rather uniform (Figure 5b).

### 4.2. Phase Composition and Microstructure of the Product of Two-Stage Mechanical Alloying of the (Cu+Fe) + Al Mixture

Mechanical alloying in the Al–Cu–Fe system was previously studied concerning the synthesis of Al_63-70_Cu_20-25_Fe_10-12_ quasicrystals [29,30,55,56]. It was stated that the Al–Cu–Fe alloy phase composition depends on the ball milling conditions [57].]. An alloy of close composition was obtained and characterized in our previous work [33]. In all those cases, the alloys were obtained via a single-stage alloying process.

After the single-stage mechanical alloying of the (Cu+Fe) + Al powder mixture [28], the following crystalline phases were found: Cu(Al) solid solution (47 wt.%), Al_4_Cu_9_ intermetallic (46 wt.%), and a non-bound α-Fe (7 wt.%). The product of the two-stage process demonstrates significant differences: X-ray amorphous phases were present, and the solid solution of aluminum in copper was formed without the intermetallic phase formation. The MS analysis revealed that iron was present in the following phases: α-Fe (32%), amorphous solid solution (FeAl)_am_ (59%), and spinel FeAl_2_O_4_ (9%). The latter is essential for application in catalysis.

It should be noted that MS allows balancing the iron-containing phases only. So, a full agreement between the quantitative analysis by MS and XRD cannot be expected. According to the XRD, the major component of the product of two-stage mechanical alloying of (Cu+Fe) + Al is a Cu(Al) solid solution. It can be inferred that the interaction with aluminum leads to the decomposition of the first-stage-synthesized solid solution of iron in copper. A fraction of this solid solution reacts with aluminum and forms an amorphous X-ray phase, whereas another fraction is consumed to form a crystalline phase.

A comparative analysis of the XRD and MS results allowed looking deeper into the mechanisms of the alloying processes during milling of the (Cu+Fe) + Al mixture. The interaction of aluminum with the Cu(Fe) solid solution according to Equation (2) leads to the formation of two types of solid solutions:

Al + Cu(Fe) → Cu(Al) + Fe(Al)_am_ (2)

Reaction (3) is also possible:

Al + Cu(Fe) → Cu(FeAl)_am_ (3)

Both MS (see inset in Figure 3) and TEM point to the presence of an oxide phase with a spinel structure. Oxidation was observed during the mechanical alloying of Cu and Al powders in [28]. In the present work, the following reactions are possible:

Cu + O_2_ → CuO (4)

CuO + Fe, Al → FeO_x_, CuFeO_y_, FeAlO_z_ (5)

These reactions can lead to the formation of the oxide phases, including those with spinel structures.

The comparison of the single- and two-stage alloying processes reveals different phase formation sequences. The intermediate products (Al_2_Cu and Al_4_Cu_9_ intermetallics) observed in the single-stage alloying process (ball milling of (Cu+Fe) + Al) did not form in two-stage alloying. In the two-stage process, a crystalline Cu–Al solid solution formed upon decomposition of a Cu–Fe solid solution.

Another interesting aspect of the alloying process is the formation of a mesoporous structure in the particles formed by two-stage alloying (Figure 6a and Figure 9a). The presence of a mesoporous structure has been confirmed by a high specific surface area of the ternary alloy (~0.9 m^2^/g). The specific surface area of non-porous particles 5–10 μm in size (Figure 8) should be only 0.1–0.2 m^2^/g. Previously, the formation of pores in metallic particles was attributed to chemical reactions with other metals [58,59]. In our case, the pores are more likely to form via fracturing and welding, as described in [45]. This phenomenon will be the subject of future investigations.

## 5. Conclusions

33Al–45Cu–22Fe (at.%) powders were prepared by two-stage mechanical alloying using high-energy ball milling. The powders were studied by XRD, MS, TEM, and EDX.. At the first stage, a Cu+Fe powder mixture was mechanically milled; at the second stage, Al was added to the alloy, and milling was continued. According to XRD, after 90 min of milling of the Cu+Fe mixture (the end of the first stage), no crystalline iron was found. A large part of α-Fe may be “invisible” by XRD due to fluorescence. The main product forming at the first stage is a metastable crystalline solid solution of iron in fcc copper, in which Fe atoms are distributed mainly in the form of clusters. The use of high-energy milling instead of conventional (low-energy) milling allows significantly reducing the time required for the alloy formation.

At the second stage, the metastable solid solution of iron in copper transforms into a crystalline Cu(Al) solid solution and X-ray amorphous Fe-containing phases. It was shown that the products of the two-stage alloying process differ from those of single-stage alloying. In contrast to single-stage alloying (ball milling of (Cu+Fe) + Al), the intermediate products, which are Al_2_Cu and Al_4_Cu_9_ intermetallics, do not form in the two-stage alloying process. Therefore, a Cu(Al) solid solution forms upon decomposition of a metastable Cu(Fe) solid solution. The products of the two-stage alloying of the 33Al–45Cu–22Fe mixture possess a mesoporous structure and also contain an oxide spinel. The formation of X-ray amorphous metastable structures and mesopores is indicative of the high chemical reactivity of the alloy product, which are desirable characteristics for catalysts and will be addressed in future studies.

## Figures and Tables

**Table 1:** Lattice parameters, crystallite sizes <D> and phase ratios Cu(Fe):Fe for the 67Cu–33Fe powders milled for 0–90 min.

Sample (Milling Time) [min]	Cu, a [Å]	Cu, <D> [nm]	α-Fe, a [Å]	α-Fe, <D> [nm]	Cu(Fe):Fe [wt.%]
Cu+Fe (0)	3.615 (1)	>100	2.866 (1)	>100	70:30
Cu+Fe (15)	3.619 (1)	19	2.869 (1)	25	77:23
Cu+Fe (30)	3.619 (1)	11	2.869 (1)	12	79:21
Cu+Fe (45)	3.626 (1)	9.5	2.871 (1)	9.5	85:15
Cu+Fe (60)	3.630 (1)	9.5	2.870 (1)	9.0	92:8
Cu+Fe (75)	3.632 (1)	10.0	2.868 (2)	10.0	97:3
Cu+Fe (90)	3.634 (1)	10.0	-	-	100:0
f					
α-Fe (0)	-	-	2.867 (1)	>100	0:100
α-Fe, (ICDD, PDF#06-0696)	-	-	2.8664	-	-
Cu, (ICDD, PDF#04-0836)	3.615	-	-	-	-

**Table 2:** Hyperfine parameters of the Mössbauer spectra were recorded from the powders *.

Sample (Milling Time) [min]	State of Iron	Area [%]	δ [mm·s^−1^]	∆ [mm·s^−1^]	B_hf_ [T]
Fe (0)	α-Fe	100	0.000 (1)	0.002 (1)	33.10 (1)
Cu+Fe (30)	α-Fe	100	0.001 (1)	0.003 (2)	33.10 (2)
Cu+Fe (60)	α-Fe	90	0.002 (2)	0.001 (2)	33.03 (3)
	Fe “fcc”	10	0.24 (4)	-	-
Cu+Fe (90)	α-Fe	79	0.003 (2)	−0.001 (3)	32.88 (3)
	Fe “fcc”	21	0.18 (2)	-	-
(Cu+Fe) + Al (24) **	α-Fe	32	0.005 (7)	−0.004 (7)	32.99 (6)
	Fe(Al)_am_	59	0.15 (5)	0.45 (6)	-
	FeAl_2_O_4_	9	1.00 (6)	0.77 (8)	-

* δ is the isomer shift reflecting the chemical neighborhood of the resonant nucleus, ∆ is the measure of the distortion of the nearest neighborhood with respect to a cubic one, B_hf_—magnetic hyperfine field [36]. ** The sample made by two-stage mechanical alloying will be discussed in the respective section below.

**Table 3:** Cu and Fe contents in flat projections are marked in Figure 6a. The linewidth is 130 eV.

Element	[keV]	Projection (1) [at.%]	Projection (2) [at.%]	Projection (3) [at.%]
Fe K	6.398	68.9	17.2	68.6
Cu K	8.040	31.1	82.8	31.4

**Table 4:** The atomic concentration of elements in the Cu+Fe mixture after 90 min of milling from regions shown in Figure 6c.

Element	keV	Area (1) [at.%]	Area (2) [at.%]
Fe K	6.398	6.6	23.6
Cu K	8.040	93.4	76.4

**Table 5:** Atomic percentages of Al, Cu and Fe in regions marked in Figure 8b,c.

Element	[keV]	Region (1) [at.%]	Region (2) [at.%]	Region (3) [at.%]	Region (4) [at.%]	Total, [at.%]
Al K	1.486	4.1	3.2	8.1	88.93	3.59
Fe K	6.398	27.7	31.5	26.1	4.18	28.70
Cu K	8.040	68.2	65.3	65.8	6.89	66.71

## Data Availability

The data presented in this study are available on request.

## References

[1] Tikhov S.F., Minyukova T.P., Valeev K.R., Cherepanova S.V., Salanov A.N., Kaichev V.V., Saraev A.A., Andreev A.S., Lapina O.B., Sadykov V.A. (2017). Design of Micro-Shell Cu–Al Porous Ceramometals as Catalysts for the Water–Gas Shift Reaction. RSC Adv..

[2] Lin X., Zhang Y., Yin L., Chen C., Zhan Y., Li D. (2014). Characterization and Catalytic Performance of Copper-Based WGS Catalysts Derived from Copper Ferrite. Int. J. Hydrog. Energy.

[3] Shechtman D., Blech I., Gratias D., Cahn J.W. (1984). Metallic Phase with Long-Range Orientational Order and No Translational Symmetry. Phys. Rev. Lett..

[4] Kelhar L., Ferčič J., Maček-Kržmanc M., Zavašnik J., Šturm S., Koželj P., Kobe S., Dubois J.-M. (2018). The Role of Fe and Cu Additions on the Structural, Thermal and Magnetic Properties of Amorphous Al-Ce-Fe-Cu Alloys. J. Non. Cryst. Solids.

[5] Mitka M., Kalita D., Góral A., Lityńska-Dobrzyńska L. (2020). The Effect of Transition Metals on Quasicrystalline Phase Formation in Mechanically Alloyed Al65Cu20Fe15 Powder. Arch. Metall. Mater..

[6] Zhu L., Soto-Medina S., Cuadrado-Castillo W., Hennig R.G., Manuel M.V. (2020). New Experimental Studies on the Phase Diagram of the Al-Cu-Fe Quasicrystal-Forming System. Mater. Des..

[7] Ali R., Akhtar M.U., Zahoor A., Ali F., Scudino S., Shahid R.N., Tariq N., Srivastava V.C., Uhlenwinkel V., Hasan B.A. (2020). Study of Thermal and Structural Characteristics of Mechanically Milled Nanostructured Al-Cu-Fe Quasicrystals. Mater. Chem. Phys..

[8] Zahoor A., Aziz T., Zulfiqar S., Sadiq A., Ali R., Shahid R.N., Tariq N., Shah A., Shehzad K., Ali F. (2020). Antimicrobial Behavior of Leached Al–Cu–Fe-Based Quasicrystals. Appl. Phys. A Mater. Sci. Process..

[9] Mishra S.S., Yadav T.P., Singh S.P., Singh A.K., Shaz M.A., Mukhopadhyay N.K., Srivastava O.N. (2020). Evolution of Porous Structure on Al–Cu–Fe Quasicrystalline Alloy Surface and Its Catalytic Activities. J. Alloys Compd..

[10] Awasthi K., Yadav T.P., Srivastava O.N. (2018). Growth of Carbon Nanotubes on Icosahedral Quasicrystalline Al–Cu–Fe Alloy. Adv. Sci. Eng. Med..

[11] Husem F., Tezel F.M., Turan M.E. (2019). Influence of Aging on Mechanical Properties, Wear and Residual Stress of a Heusler Al-Cu-Fe Alloy. Mater. Test..

[12] Lan X., Wang H., Sun Z., Jiang X. (2019). Al–Cu–Fe Quasicrystals as the Anode for Lithium Ion Batteries. J. Alloys Compd..

[13] Tsetlin M.B., Teplov A.A., Belousov S.I., Chvalun S.N., Golovkova E.A., Krasheninnikov S.V., Golubev E.K., Pichkur E.B., Dmitryakov P.V., Buzin A.I. (2018). Composite Material Based on Polytetrafluoroethylene and Al–Cu–Fe Quasi-Crystal Filler with Ultralow Wear: Morphology, Tribological, and Mechanical Properties. J. Surf. Investig. X Ray Synchrotron Neutron Tech..

[14] Tanabe T., Kameoka S., Tsai A.P. (2006). A Novel Catalyst Fabricated from Al–Cu–Fe Quasicrystal for Steam Reforming of Methanol. Catal. Today.

[15] Tanabe T., Kameoka S., Tsai A.P. (2011). Evolution of Microstructure Induced by Calcination in Leached Al–Cu–Fe Quasicrystal and Its Effects on Catalytic Activity. J. Mater. Sci..

[16] Borshch V.N., Dement’eva I.M., Khomenko N.Y. (2019). Supported Polymetallic Catalysts by Self-Propagating Surface Synthesis. Int. J. Self Propagating High Temp. Synth..

[17] Borshch V.N., Zhuk S.Y., Sachkova N.V. (2018). Activation of the Surface of Polymetallic Carriers by the Formation of Intermediate Intermetallic Phases. Kinet. Catal..

[18] Borshch V.N., Pugacheva E.V., Zhuk S.Y., Smirnova E.M., Demikhova N.R., Vinokurov V.A. (2020). Hydrogenation of CO_2_ on the Polymetallic Catalysts Prepared by Self-Propagating High-Temperature Synthesis. Russ. Chem. Bull..

[19] Popov A.A., Shubin Y.V., Bauman Y.I., Plyusnin P.E., Mishakov I.V., Sharafutdinov M.R., Maksimovskiy E.A., Korenev S.V., Vedyagin A.A. (2020). Preparation of Porous Co-Pt Alloys for Catalytic Synthesis of Carbon Nanofibers. Nanotechnology.

[20] Mishakov I.V., Kutaev N.V., Bauman Y.I., Shubin Y.V., Koskin A.P., Serkova A.N., Vedyagin A.A. (2020). Mechanochemical Synthesis, Structure, and Catalytic Activity of Ni-Cu, Ni-Fe, and Ni-Mo Alloys in the Preparation OF Carbon Nanofibers During the Decomposition of Chlorohydrocarbons. J. Struct. Chem..

[21] Rudneva Y.V., Shubin Y.V., Plyusnin P.E., Bauman Y.I., Mishakov I.V., Korenev S.V., Vedyagin A.A. (2019). Preparation of Highly Dispersed Ni1-XPdx Alloys for the Decomposition of Chlorinated Hydrocarbons. J. Alloys Compd..

[22] Kenzhin R.M., Bauman Y.I., Volodin A.M., Mishakov I.V., Zaikovskii V.I., Vedyagin A.A. (2019). Microscopic Studies on the Polymers Decomposition in a Closed Volume at Elevated Temperatures in the Presence of Bulk NiCr Alloy. SN Appl. Sci..

[23] Theofanidis S.A., Poelman H., Marin G.B., Galvita V.V. (2019). How Does the Surface Structure of Ni-Fe Nanoalloys Control Carbon Formation During Methane Steam/Dry Reforming?. Advanced Nanomaterials for Catalysis and Energy.

[24] Vaidya M., Muralikrishna G.M., Murty B.S. (2019). High-Entropy Alloys by Mechanical Alloying: A Review. J. Mater. Res..

[25] Tebeta R.T., Fattahi A.M., Ahmed N.A. (2020). Experimental and Numerical Study on HDPE/SWCNT Nanocomposite Elastic Properties Considering the Processing Techniques Effect. Microsyst. Technol..

[26] Tikhov S.F., Minyukova T.P., Valeev K.R., Cherepanova S.V., Salanov A.N., Shtertser N.V., Sadykov V.A. (2019). Design of Ceramometal CuFeAlOx/CuFeAl Composites and Their Catalytic Potential for Water Gas Shift Reaction. Mater. Chem. Phys..

[27] Tikhov S.F., Sadykov V.A., Valeev K.R., Salanov A.N., Cherepanova S.V., Bespalko Y.N., Ramanenkau V.E., Piatsiushyk Y.Y., Dimov S.V. (2015). Preparation of Porous Ceramometal Composites through the Stages of Mechanical Activation and Hydrothermal Partial Oxidation of Me–Al Powders. Catal. Today.

[28] Dudina D.V., Lomovsky O.I., Valeev K.R., Tikhov S.F., Boldyreva N.N., Salanov A.N., Cherepanova S.V., Zaikovskii V.I., Andreev A.S., Lapina O.B. (2015). Phase Evolution during Early Stages of Mechanical Alloying of Cu–13wt.% Al Powder Mixtures in a High-Energy Ball Mill. J. Alloys Compd..

[29] Huttunen-Saarivirta E. (2004). Microstructure, Fabrication and Properties of Quasicrystalline Al–Cu–Fe Alloys: A Review. J. Alloys Compd..

[30] Tomilin I.A., Kaloshkin S.D., Tcherdyntsev V.V. (2006). Enthalpy of Formation of Quasicrystalline Phase and Ternary Solid Solutions in the Al-Fe-Cu System. Rare Met..

[31] Fleury E., Lee S.M., Choy G., Kim W.T., Kim D.H. (2001). Comparison of Al-Cu-Fe Quasicrystalline Particle Reinforced Al Composites Fabricated by Conventional Casting and Extrusion. J. Mater. Sci..

[32] Laplanche G., Joulain A., Bonneville J., Schaller R., El Kabir T. (2010). Microstructures and Mechanical Properties of Al-Base Composite Materials Reinforced by Al–Cu–Fe Particles. J. Alloys Compd..

[33] Tikhov S.F., Valeev K.R., Salanov A.N., Cherepanova S.V., Boldyreva N.N., Zaikovskii V.L., Sadykov V.A., Dudina D.V., Lomovsky O.I., Romanenkov V.E. (2018). Phase Formation during High-Energy Ball Milling of the 33Al-45Cu-22Fe (at.%) Powder Mixture. J. Alloys Compd..

[34] Yavari A.R., Desré P.J., Benameur T. (1992). Mechanically Driven Alloying of Immiscible Elements. Phys. Rev. Lett..

[35] Agüero O.E., Socolovsky L.M., Torriani I.L. (2004). Crystallite Size and Strain Study of a Nanostructured Fe-Cu Alloy from Diffraction Profile Analysis. J. Metastable Nanocrystalline Mater..

[36] Socolovsky L.M., Sánchez F.H. (2004). Concentration Dependence of Hyperfine Parameters of Fe-Cu Alloys. J. Metastable Nanocrystalline Mater..

[37] Raghavan V. (2005). Al-Cu-Fe (Aluminum-Copper-Iron). J. Phase Equilibria Diffus..

[38] Proto Unmasking Microabsorption: Why Fluorescence Suppression in Powder XRD Does More Harm Than Good. https://www.azom.com/article.aspx?ArticleID=20003.

[39] Dunlap R.A., Lloyd D.J., Christie I.A., Stroink G., Stadnik Z.M. (1988). Physical Properties of a Rapidly Quenched Al-Fe Alloys. J. Phys. F Met. Phys..

[40] Dunlap R.A., Dahn J.R., Eelman D.A., MacKay G.R. (1998). Microstructure of Supersaturated Fcc Al–Fe Alloys: A Comparison of Rapidly Quenched and Mechanically Alloyed Al98Fe2. Hyperfine Interact..

[41] Dézsi I., Szűcs I., Sváb E. (2000). Mössbauer Spectroscopy of Spinels. J. Radioanal. Nucl. Chem..

[42] Miedema A.R., de Châtel P.F., de Boer F.R. (1980). Cohesion in Alloys—Fundamentals of a Semi-Empirical Model. Phys. B+C.

[43] Duwez P., Willens R.H., Klement W. (1960). Metastable Solid Solutions in the Gallium Antimonide-Germanium Pseudobinary System. J. Appl. Phys..

[44] Sumiyama K., Yoshitake Y., Nakamura Y. (1985). Thermal Stability of High Concentration Fe-Cu Alloys Produced by Vapor Quenching. Acta Metall..

[45] Benjamin J. (1976). Mechanical Alloying. Sci. Am..

[46] Grigorieva T.F., Barinova A.P., Lyakhov N.Z. (2001). Mechanochemical Synthesis of Intermetallic Compounds. Russ. Chem. Rev..

[47] Kaloshkin S.D., Tomilin I.A., Andrianov G.A., Baldokhin U.V., Shelekhov E.V. (1996). Phase Transformations and Hyperfine Interactions in Mechanically Alloyed Fe-Cu Solid Solutions. Mater. Sci. Forum.

[48] Barro M.J., Navarro E., Agudo P., Hernando A., Crespo P., Garcia Escorial A. (1996). Structural Evolution during Milling of Diluted Solid Solutions of Fe-Cu. Mater. Sci. Forum.

[49] Uenishi K., Kobayashi K.F., Nasu S., Hatano H., Ishihara K.N. (1992). Mechanical Alloying in the Fe-Cu System. Zeitschrift Met..

[50] Eckert J., Holzer J.C., Johnson W.L. (1993). Thermal Stability and Grain Growth Behavior of Mechanically Alloyed Nanocrystalline Fe-Cu Alloys. J. Appl. Phys..

[51] Macrí P.P., Rose P., Banda D.E., Cowlam N., Principi G., Enzo S. (1995). A Study of the Consumption of Iron during the Mechanical Alloying of the Cu-Fe Immiscible System. Mater. Sci. Forum.

[52] Elkalkouli R., Chartier P., Dinhut J.F. (1995). Structure and Thermal Stability of CuCo and CuFe Alloys Prepared by Mechanical Alloying. Mater. Sci. Forum.

[53] Dunlap R.A., Eelman D.A., Mackay G.R. (1998). Fe Clustering in f.c.c. Cu-Fe Alloys Prepared by Mechanical Alloying. J. Mater. Sci. Lett..

[54] Huang X., Mashimo T. (1999). Metastable BCC and FCC Alloy Bulk Bodies in Fe–Cu System Prepared by Mechanical Alloying and Shock Compression. J. Alloys Compd..

[55] Mukhopadhyay N.K., Yadav T.P., Srivastava O.N. (2002). An Investigation on the Transformation of the Icosahedral Phase in the Al-Fe-Cu System during Mechanical Milling and Subsequent Annealing. Philos. Mag. A.

[56] Bokhonov B.B. (2008). Mechanical Alloying and Self-Propagating High-Temperature Synthesis of Stable Icosahedral Quasicrystals. J. Alloys Compd..

[57] Srinivas V., Barua P., Murty B. (2000). On Icosahedral Phase Formation in Mechanically Alloyed Al70Cu20Fe10. Mater. Sci. Eng. A.

[58] Tikhov S.F., Usoltsev V.V., Salanov A.N., Tsybulya S.V., Chesalov Y.V., Kustova G.N., Sadykov V.A., Golubkova G.V., Lomovskii O.I. (2010). Design of Composite Porous Cermets Synthesized by Hydrothermal Treatment of CrAl Powder Followed by Calcination. J. Mater. Sci..

[59] Usoltsev V.V., Tikhov S.F., Salanov A.N., Sadykov V.A., Golubkova G.V., Lomovskii O.I. (2013). Properties of Porous FeAlO y /FeAl x Ceramic Matrix Composite Influenced by Mechanical Activation of FeAl Powder. Bull. Mater. Sci..

